# Supplementary material for: Endometrial Epithelial ARID1A Is Required for Uterine Immune Homeostasis during Early Pregnancy
Source: Int J Mol Sci. 2022 May 28;23(11):6067. doi: 10.3390/ijms23116067 (PMC9181301; doi:10.3390/ijms23116067)
Supplement: Supplementary file 1 [file ijms-23-06067-s001.zip › ijms-1668732-supplementary.pdf]

International Journal of *Molecular Sciences*

Supplementary Materials

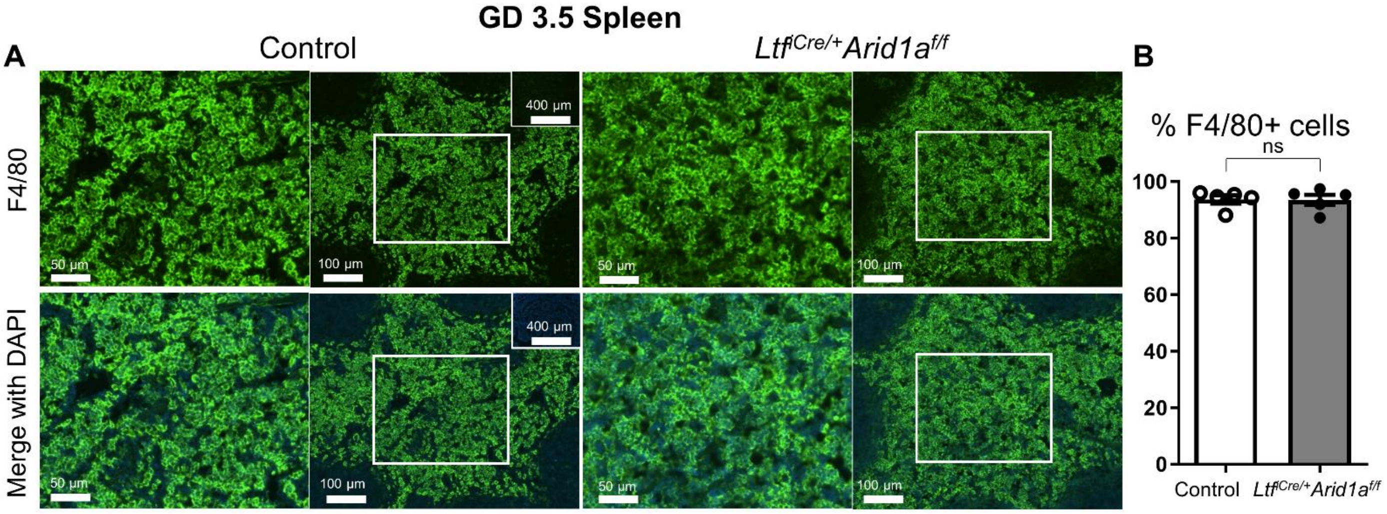

Supplementary Figure S1. F4/80+ macrophage numbers are unchanged in the spleen tissue of *Ltf*$^{iCre/+}$*Arid1a*$^{f/f}$ mice at GD 3.5. (A) Representative images of F4/80 immunofluorescence (green) counterstained with DAPI (blue) in control (left) and *Ltf*$^{iCre/+}$*Arid1a*$^{f/f}$ (right) mouse spleen sections at GD 3.5 (n=5/genotype) Insets: no primary antibody negative controls. (B) The percentage of F4/80-positive spleen cells was counted in representative red pulp fields of approximately 500 cells per sample of control (n=5, empty bar) and *Ltf*$^{iCre/+}$*Arid1a*$^{f/f}$ (n=5, grey bar) uteri at GD3.5. The graph represents the mean ± SEM. ns, p>0.05. Abbreviations: *Arid1a*, AT-rich interaction domain 1A; F4/80, EGF-like module-containing mucin-like hormone receptor-like 1; GD, gestation day; *Ltf*, lactoferrin.

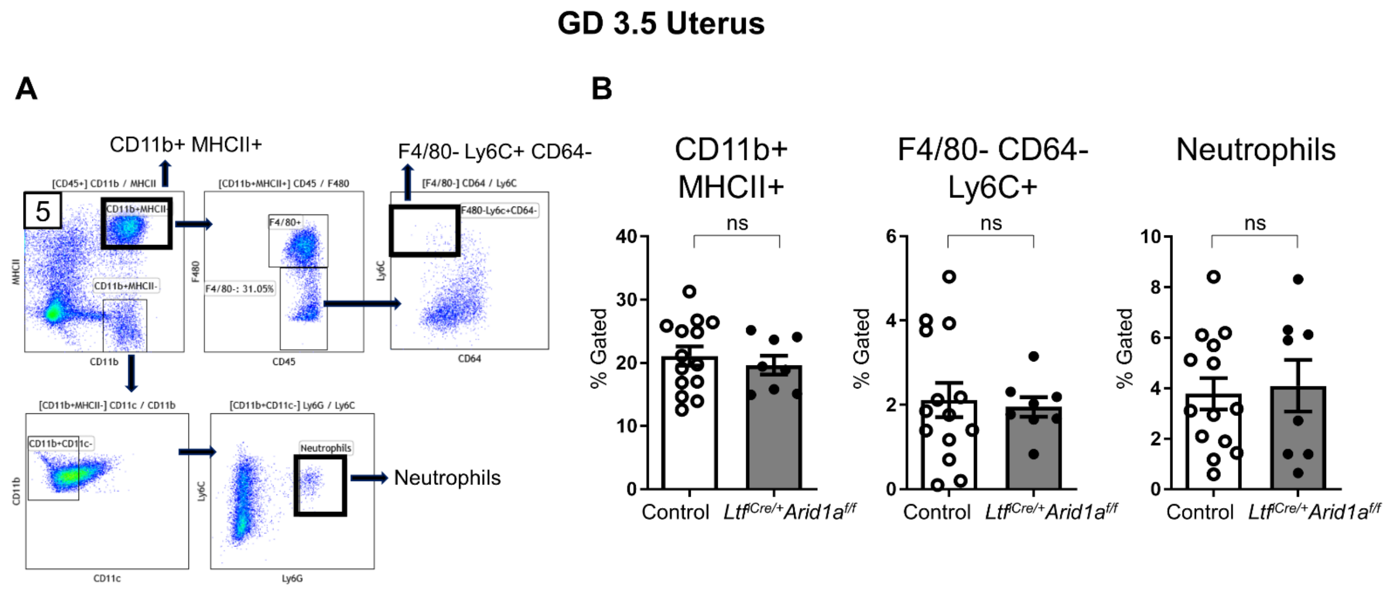

Supplementary Figure S2. No change was detected in the proportions of CD11b+ MHCII+ cells, F4/80- CD64- Ly6C+ cells, or Neutrophils in *Ltf*$^{iCre/+}$*Arid1a*$^{f/f}$ uteri at GD 3.5. (A) The flow gating strategy to identify CD11b+ MHCII+ cells, F4/80- CD64- Ly6C+ cells, and Neutrophils in the mouse uterus is shown. (B) The percentage Gated proportion of CD11b+ MHCII+ cells (left), F4/80- CD64- Ly6C+ cells (middle), and neutrophils (right) in control (n=14, empty bar) and *Ltf*$^{iCre/+}$*Arid1a*$^{f/f}$ (n=8, grey bar) uteri at GD3.5 is shown. The graphs represent the mean ± SEM. ns, p>0.05. Abbreviations: *Arid1a*, AT-rich interaction domain 1A; CD11b, cluster of differentiation molecule 11B; CD64, cluster of differentiation 64; F4/80, EGF-like module-containing mucin-like hormone receptor-like 1; GD, gestation day; *Ltf*, lactoferrin; Ly6c, lymphocyte antigen 6C; MHCII, major histocompatibility complex class II.

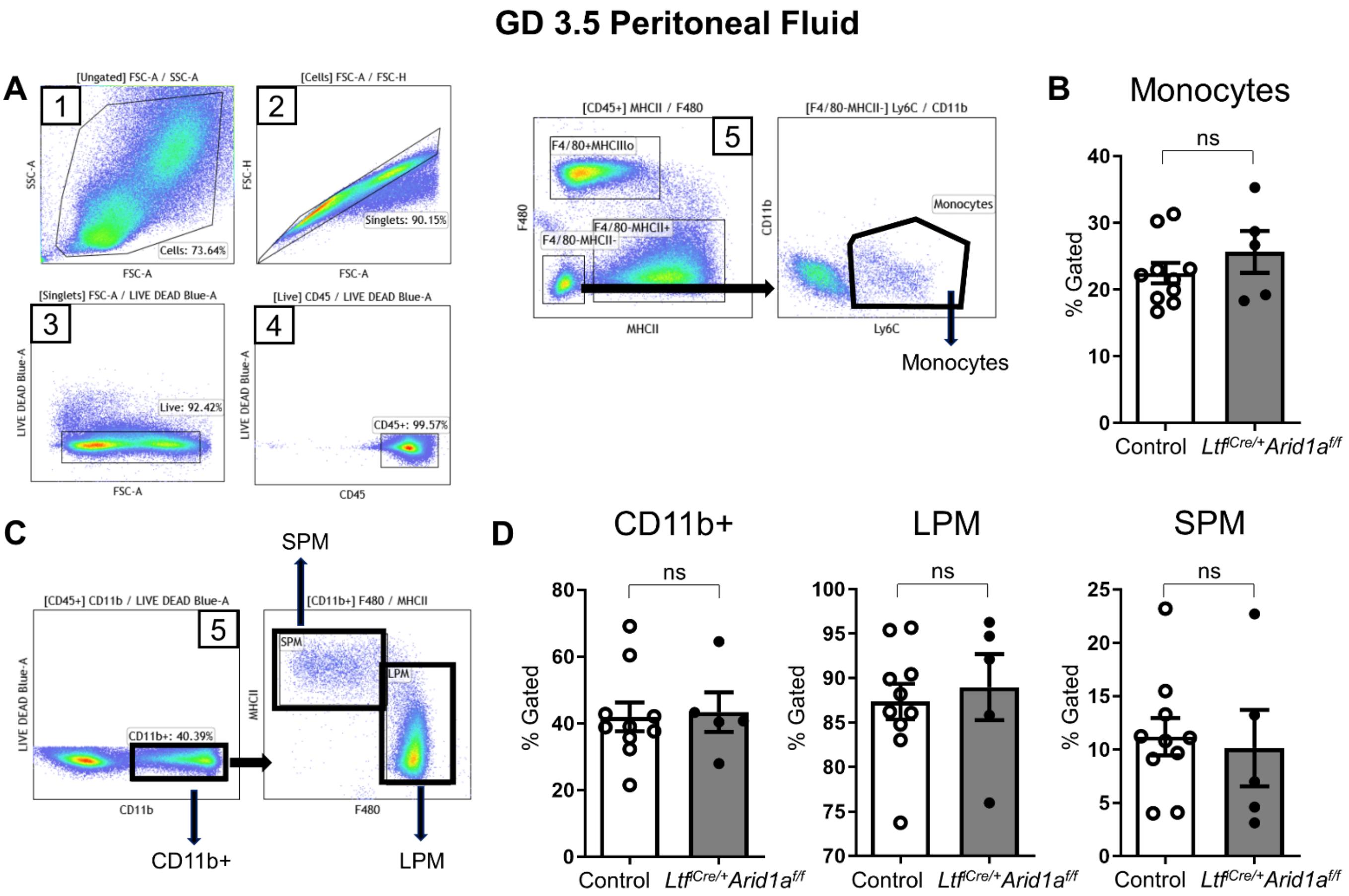

Supplementary Figure S3. No change was detected in proportions of CD11b+ cells, large peritoneal macrophages (LPM), small peritoneal macrophages (SPM), or Ly6C+ monocytes in *Ltf*$^{iCre/+}$*Arid1a*$^{f/f}$ peritoneal fluid at GD3.5. (A) The flow gating strategy to identify Ly6C+ monocytes in the peritoneal fluid is shown. (B) The percentage Gated proportion of Ly6C+ monocytes in control (n=14, empty bar) and *Ltf*$^{iCre/+}$*Arid1a*$^{f/f}$ (n=8, grey bar) peritoneal fluid at GD3.5 is shown. (C) The flow gating strategy to identify CD11b+ cells, LPM, and SPM in the peritoneal fluid is shown. (D) The percentage Gated proportion of CD11b+ cells (left), LPM (middle), and SPM (right) in control (n=14, empty bar) and *Ltf*$^{iCre/+}$*Arid1a*$^{f/f}$ (n=8, grey bar) peritoneal fluid at GD3.5 is shown. The graphs represent the mean ± SEM. ns, p>0.05. Abbreviations: *Arid1a*, AT-rich interaction domain 1A; CD11b, cluster of differentiation molecule 11B; GD, gestation day; LPM, large peritoneal macrophages; *Ltf*, lactoferrin; SPM, small peritoneal macrophages.

Supplementary Table S1. The differentially expressed gene list from RNA-sequencing of the GD 3.5 *Ltf*$^{iCre/+}$*Arid1a*$^{f/f}$ compared to control uterus contains 4,181 genes. There were 2,007 decreased and 2,174 increased significantly differentially expressed genes meeting the threshold of FDR<0.05, corrected for multiple testing by independent hypothesis weighting. This table of differentially expressed genes in the *Ltf*$^{iCre/+}$*Arid1a*$^{f/f}$ uterus at GD3.5 is available as a .xlsx file.
